# Dupilumab-Associated Angioedema in an Adult: A Case Report of an Adverse Event and Literature Review

**DOI:** 10.7759/cureus.41406

**Published:** 2023-07-05

**Authors:** Trevor Hodson, Divya Shah, Duane Wong

**Affiliations:** 1 Internal Medicine, University of Arizona College of Medicine, Phoenix, USA; 2 Allergy and Immunology, Arizona Allergy Associates, Phoenix, USA

**Keywords:** new drug reaction, nasal polyposis, dupilumab, facial angioedema, drug-induced angioedema

## Abstract

Dupilumab is a monoclonal antibody that targets interleukin (IL)-4 and IL-13 for use in moderate to severe eczema, asthma, and nasal polyposis. Our case report presents a 47-year-old woman with a history of nasal polyposis who developed angioedema after being treated with dupilumab for recurrent polyposis. She tolerated her first dose of dupilumab without reaction, but 10 days after her second injection, she developed swelling of the lips and forehead. She was treated with steroids with partial resolution. She received two further doses, which followed similar courses before dupilumab was discontinued. To the best of the authors' knowledge, this is the first report of dupilumab-associated angioedema in an adult. This report may be instructional for prescribers providing patients with anticipatory guidance or evaluating otherwise unexplained angioedema.

## Introduction

Angioedema is a clinical entity characterized by increased permeability of the blood vessels in the deep dermis and subcutaneous tissues resulting in tissue swelling [1]. Its etiologies can be broadly broken into two categories: mast cell mediated or induced via the bradykinin pathway [2]. Mast cell-mediated angioedema features an immunoglobulin E (IgE) response releasing histamine, resulting in hives and swelling within minutes to hours of exposure. The natural bradykinin pathway can result in angioedema when dysregulation occurs via missing inhibitors due to genetics, drugs, consumptive diseases, or autoantibodies [3].

Dupilumab is a human monoclonal antibody that inhibits interleukin (IL)-4 and IL-13 signaling by specifically binding to the IL-4 receptor alpha subunit, which is shared by the IL-4 and IL-13 receptor complexes. By blocking these receptors, dupilumab inhibits the IL4 and IL-13 pathways, thus preventing their downstream effects, which includes the release of proinflammatory cytokines, chemokines, and IgE, all of which can play a role in the type 2 (T2) inflammatory process [4]. Dupilumab is thus frequently prescribed for diseases in which a component of the T2 inflammation is involved, such as skin diseases like atopic dermatitis, allergic contact dermatitis, and nummular eczema and respiratory diseases like allergic rhinitis, allergic bronchopulmonary aspergillosis, and chronic eosinophilic pneumonia [5]. As the overall prevalence of dupilumab use grows, it is expected that the medical community will gain a greater understanding of its benefits and potential adverse events. To date, only a single case of angioedema associated with dupilumab has been reported in a child, but never in an adult [6-7]. This case was previously presented as a poster at the 2023 American Academy of Allergy, Asthma & Immunology Annual Meeting on February 25, 2023 and then published as a brief abstract in the supplement of the February 2023 edition of the *Journal of Allergy and Clinical Immunology*.

## Case presentation

**Figure 1:**
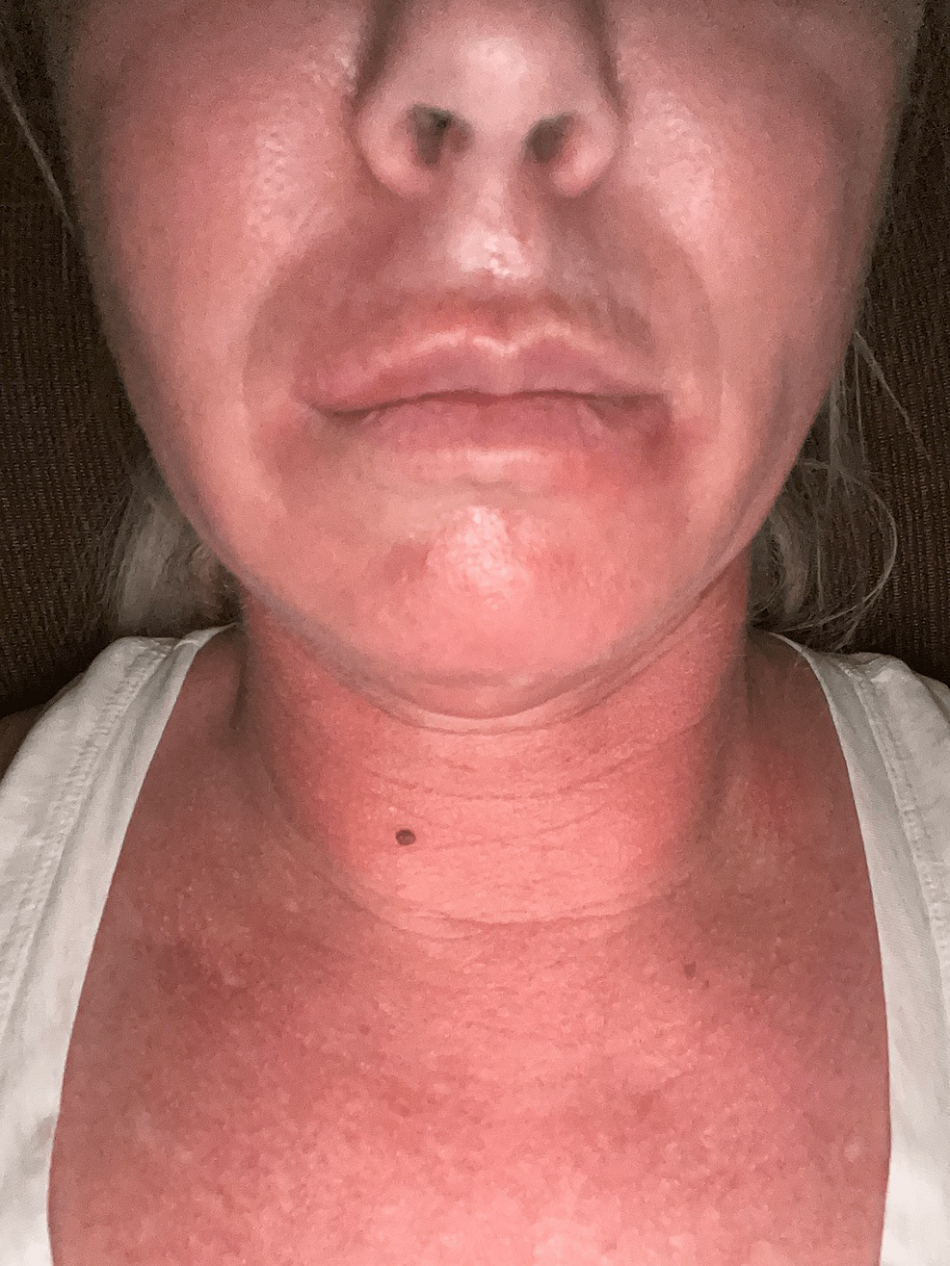
Facial swelling 10 days after the second dupilumab injection.

Our case report presents a 47-year-old white woman with a past medical history of nasal polyposis who developed angioedema after being treated with dupilumab for recurrent polyposis. The patient was initially seen at an otolaryngology office for complaints of persistent congestion. She had been treated with polypectomy 10 years prior with only temporary relief before a return of her polyposis. It was at this office that she was given her first dose of dupilumab without reaction. Ten days after her second injection, she developed swelling of the lips and forehead (Figure 1).

The patient experienced no erythema or perceptible rash and no respiratory distress. She was treated with a corticosteroid burst with near-complete resolution of swelling. Following resolution, she was then given a third dose of dupilumab for nasal polyposis, and one week later, her swelling recurred. She was again treated with steroids without complete resolution of swelling. Despite these symptoms, she was still given her scheduled fourth dose and suffered worsening swelling again one week following injection. Dupilumab was then discontinued, and the patient’s swelling decreased over the next two months, during which time she was referred to the author’s practice for further evaluation. Laboratory workup revealed normal C3, C4, and C1 esterase inhibitor levels, and the C1 esterase inhibitor function was also normal at 91% (Table 1). Given the consistent swelling of the lips and forehead on three separate occasions following injection, the decision was made to forgo further administration of dupilumab, and the patient was started on a fluticasone nasal spray with improvement of her nasal symptoms. Since discontinuing dupilumab, the patient has not had any further swelling at 10 months follow-up and is being considered for polypectomy for further relief of her nasal polyposis.

**Table 1:** Key laboratory values two months after the final dupilumab dose mg: miligrams; dL: deciliter; IU: international units; U: units

Lab name	Lab value	Reference range
C3 complement component	108 mg/dL	90-180 mg/dL
C4 complement component	22 mg/dL	16-47 mg/dL
C1 esterase inhibitor protein	32 mg/dL	21-39 mg/dL
Immunoglobulin E	102 IU/ml	<100 IU/ml
C1 esterase inhibitor function	91%	>68%
C1Q complement component	6.5 mg/dL	5-8.6 mg/dL
Total complement	>60 U/ml	31-60 U/ml

## Discussion

As the use of dupilimab in clinical practice increases, it will be crucial for clinicians to be able to forewarn and identify potential side effects, including dupilumab-associated angioedema. Knowledge of this potential side effect is currently limited. A thorough search of an online medical database (PubMed) was performed using the keywords “Angioedema” AND “Dupilumab" OR “Swelling” AND “Dupilumab,” and only a single case report was found, which reported angioedema in association with dupilumab. This case, however, was in a child treated for recalcitrant atopic dermatitis. In that case, the patient developed pruritic hives, swelling of the cheeks and lips, and gastrointestinal cramping one week following dupilumab injection, which improved with oral antihistamines. This contrasts the authors' report in that the adult treated with dupilumab developed no rashes and the primary symptom was facial and labial swelling.

We believe this to be the first reported case of dupilumab-associated angioedema in an adult. Perhaps most convincing about this case is the fact that the patient experienced the same reaction replicated over three separate doses. Of particular interest is the mechanism whereby this reaction could have occurred. Given the patient's normal complement levels and normal C1 inhibitor functionality as well as the lack of prior or subsequent episodes, hereditary angioedema as a cause of this patient's swelling remains unlikely. Transient increases in eosinophils following dupilumab injection have been noted in the literature [8]. One theory is whether patients with subclinical hypereosinophilia might experience rising eosinophils following injection resulting in angioedema. Further research will be needed to determine if such an association exists, and subsequent screening of eosinophil levels could be considered as a prerequisite to therapy. Unfortunately, no blood counts were completed at the time of this patient's adverse event. Further research will be needed to further elucidate whether dupilumab interacts with the bradykinin or histamine-mediated pathways of angioedema in select patients.

## Conclusions

To the best of the authors' knowledge, this is the first reported case of dupilumab-associated angioedema in an adult. Further monitoring will be needed to evaluate whether these reactions are replicated in future cases. While awaiting future reports, prescribers providing anticipatory guidance should be aware of this possible side effect, and clinicians may consider adding dupilumab to their differential diagnoses for otherwise unexplained angioedema.
